# A mouse model for interstitial cystitis/painful bladder syndrome based on APF inhibition of bladder epithelial repair: a pilot study

**DOI:** 10.1186/1471-2490-12-17

**Published:** 2012-06-08

**Authors:** Susan Keay, Samantha Leitzell, Ashley Ochrzcin, George Clements, Min Zhan, David Johnson

**Affiliations:** 1 Department of Medicine, University of Maryland School of Medicine, Baltimore, MD, USA; 2 Veterans Administration Maryland Health Care System, 10 North Greene Street, Room 3B-184, Baltimore, MD, 21201, USA; 3 Baltimore Research and Education Foundation, Baltimore, MD, USA; 4 Department of Epidemiology and Public Health, University of Maryland School of Medicine, Baltimore, MD, USA

**Keywords:** Interstitial cystitis, Painful bladder syndrome, Mouse model

## Abstract

**Background:**

Interstitial cystitis/painful bladder syndrome (IC/PBS) is a chronic bladder disorder with bladder epithelial thinning or ulceration, pain, urinary frequency and urgency. There is no reliably effective therapy for IC/PBS, and no generally accepted animal model for the disorder in which potential therapies can be tested. Bladder epithelial cells from IC/PBS patients make a small glycopeptide antiproliferative factor or "APF" that inhibits proliferation, decreases tight junction protein expression, increases paracellular permeability, and induces changes in gene expression of bladder epithelial cells *in vitro* that mimic abnormalities in IC/PBS patient biopsy specimens *in vivo*. We therefore determined the ability of a synthetic APF derivative to inhibit bladder epithelial repair in mice.

**Methods:**

The bladder epithelium of female CBA/J mice was stripped by transurethral infusion of 3% acetic acid, and mice were subsequently treated daily with one of three intravesical treatments [synthetic *as*-APF, inactive unglycosylated control peptide, or phosphate buffered saline carrier (PBS)] for 1–21 days. Fixed bladder sections were either stained with haematoxylin and eosin for determination of epithelial area by image analysis, or incubated with anti-uroplakin III (UPIII) or anti-zonula occludens type 1 (ZO-1) antibodies for immunofluorescence microscopy. Epithelial measurement data were analyzed by a two-way analysis of variance (ANOVA); post hoc comparisons of multiple groups were carried out using the Tukey-Kramer method.

**Results:**

Bladder epithelial repair was significantly attenuated in *as*-APF-treated mice as compared to control mice on days 3–21 (p < 0.05); the mean epithelial/total area over all measured days was also significantly lower in *as*-APF-treated mice vs. mice in either control group by post hoc analysis (p < 0.0001 for both comparisons). UPIII and ZO-1 expression was also decreased in *as*-APF-treated mice as compared to mice in either control group by day 7 (UPIII) or day 14 (ZO-1).

**Conclusions:**

This model demonstrates *in vivo* effects of *as*-APF which abrogates bladder epithelial repair and expression of UPIII and ZO-1 in CBA/J mice following transurethral acetic acid infusion. As bladder epithelial thinning, decreased UPIII expression, and decreased ZO-1 expression are histopathologic features of IC/PBS patient biopsies, this model may be useful for studying the pathophysiology of IC/PBS and the effect of potential therapies.

## Background

Interstitial cystitis/painful bladder syndrome (IC/PBS) is a chronic illness characterized by bladder epithelial thinning or ulceration, pain, urinary frequency and urgency [1-3]. The etiology of IC/PBS remains unknown, and no treatment is reliably effective. Therefore, a greater understanding of the pathogenesis of this debilitating chronic painful bladder syndrome, along with the development of animal models based on that pathogenesis, may be necessary for the development of more effective therapy(ies).

Epithelial abnormalities are indeed a cardinal finding in bladder biopsies from IC patients, with predominant histologic findings including denudation and tears in the bladder epithelium (Hunner's ulcers or glomerulations) and/or thinning of the bladder epithelium to 1–2 cell layers thick [1-5]. Although tissue from patients with Hunner's ulcers typically contains inflammatory cell infiltrates in the epithelium (or lamina propria) that often consists of T lymphocytes with or without mast cells [1,3], little inflammation is usually seen in the epithelium or submucosal interstitial tissue from the much larger number of patients without ulcers [3], indicating that inflammation in tissue superficial to the detrusor muscle is not a consistent finding in nonulcerative IC/PBS patients. However, epithelial cell proliferation and gene expression consistently appear to be abnormal in bladder tissue from IC/PBS patients *in vivo*[6-12], and abnormalities in cell proliferation and expression of most of the same genes have also been demonstrated in isolated explanted IC/PBS cells *in vitro*[13-20] with altered levels of specific cell proteins [including increased E-cadherin**,** inducible nitric oxide synthase (iNOS), plus P2X2 and P2X3 receptors; but decreased uroplakin III (UPIII), zonula occludens type 1 (ZO-1), occludin, and vimentin]. Taken together, these findings suggest an intrinsic bladder epithelial cell defect with specifically altered epithelial cell gene expression in IC/PBS patients.

To date, over 20 existing animal models of IC/PBS have been described. With the exceptions of the naturally occurring feline interstitial cystitis model and a model involving spontaneous cystitis in estrogen receptor beta-deficient mice, the other models generally involve the induction of bladder inflammation and/or epithelial damage via intravesical instillation of chemical irritants, systemic instillation of self or foreign antigens to induce an immune cell infiltrate in the bladder, or systemic viral infection to induce bladder epithelial damage [21-35]. While some of these models have been shown to express altered bladder or immune cell expression of inflammatory cytokines, only a few have been shown to exhibit abnormal expression of some of the epithelial cell proteins found to be abnormally expressed in IC/PBS patient biopsies (to date these have been limited to decreased ZO-1 in feline IC, decreased UPIII in acrolein-induced and CYP-induced cystitis, and increased iNOS in feline IC and CYP-induced cystitis); therefore, the relationship of these models to the human illness, or their utility for testing therapeutic or preventive agents for this syndrome, remains unknown.

In addition to the gene expression abnormalities noted above, we discovered that the same IC/PBS cell explants that express abnormal quantities of certain epithelial cell proteins similar to those found in IC/PBS cell biopsies also secrete a novel Frizzled 8-related glycopeptide “antiproliferative factor” (APF) [16,36,37] whose activity is also found in urine of 94-97% of patients who fulfill the symptomatic, exclusionary, and cystoscopic NIDDK criteria for ulcerative or non-ulcerative IC and 93% of patients who fulfill symptomatic and exclusionary criteria alone [38-43]. This small sialoglycopeptide (Neu5Acα2-3Galβ1-3GalNAcα-*O*-TVPAAVVVA) causes abnormalities in normal bladder epithelial cells and bladder cancer cell lines that mimic changes seen in explanted IC/PBS cells, including profoundly inhibited cell proliferation [13,16,44], increased p53 and p21 expression [44,45], altered epithelial growth factor production [16,46], and a specifically altered gene expression pattern including increased E-cadherin with decreased ZO-1, occludin, and vimentin [14,15]. APF may therefore play a role in the pathogenesis of IC/PBS by inducing these abnormalities *in vivo*.

Prior patient studies indicate that the onset of IC/PBS symptoms may be preceded by clinical evidence for a urinary tract infection [47], and previous animal studies indicate frequent shedding of the bladder epithelium as part of a response to bacterial pathogens [48]. We therefore postulated that IC/PBS may occur following bladder epithelial damage (as from bacterial cystitis or other cause) in patients who have impaired bladder epithelial repair because their epithelial cells secrete the APF toxin. With this hypothesis in mind, we performed pilot studies of a mouse model of IC/PBS based on inhibition of bladder epithelial repair by synthetic *as*-APF, using acetic acid to induce epithelial damage as previously described for a rabbit model of cystitis as well as a rat model of colitis [49,50], and determining bladder epithelial area, UPIII and ZO-1 gene expression (all previously shown to be abnormally decreased in IC/PBS patient biopsy specimens).

## Methods

### Procedure

Five to six week old female CBA/J mice (Harlan Laboratories, Frederick, MD) were anesthetized using 2-3% isoflurane in 100% oxygen via a precision vaporizer, their bladders were infused transurethrally with 50 μl of 3% acetic acid, and temporary reversible obstruction was achieved by applying collodion U.S.P. (J.T. Baker, Phillipsburg, NJ) to the external urethral meatus. The collodion was removed 1 hour later with acetone, and the mouse bladders were rinsed by transurethral infusion with sterile phosphate buffered saline (PBS) (Quality Biologicals, Inc., Gaithersburg, MD) and further infused with one of three treatments (in 50 μl total volume): 250 mM synthetic *as*-APF (PolyPeptide Laboratories, San Diego, CA), 250 mM inactive negative control peptide (PolyPeptide Laboratories), or PBS carrier alone. The bladders were then immediately obstructed for an additional 3 hours with collodion, the collodion was again removed, and the animals were returned to their cages and allowed to void until the next day. Buprenorphine (0.05-0.1 mg/kg) was administered subcutaneously to the mice every 8–12 hours for 48 hours to relieve any discomfort resulting from acetic acid treatment. The intravesical treatment infusion (with *as*-APF, peptide, or PBS) was then repeated daily for 1–21 days until sacrifice by CO_2_ inhalation. NIH guidelines for the care and use of laboratory animals for experimental procedures were followed throughout the project, and this work was approved by the Institutional Animal Care and Use Committee of the University of Maryland, Baltimore.

### Tissue fixation and staining

In anesthetized mice, bladder tissue was fixed *in situ* by infusing 50 μL of 10% buffered neutral formalin (EMD, Gibbstown, NJ) into the bladders with an infusion pump (Harvard Apparatus, Millis, MA) over 30 seconds. Bladders were then removed and fixed as a whole in 10% formalin overnight, after which they were embedded in paraffin, cut into 6 μm sections, and either stained with haematoxylin and eosin (H&E) or incubated with specific antibodies for immunofluorescence microscopy.

For immunofluorescence microscopy, sections were deparaffinized, rehydrated, and incubated in 0.1% aqueous Saponin for 1 hour, after which they were treated with Antigen Unmasking Solution (Vector Labs, Burlingame, CA) in a microwave oven for 15 minutes. Sections were then incubated with mouse anti-bovine Uroplakin III (clone 5 F161, US Biological, Swampscott Massachusetts) at 4 C overnight, followed by secondary antibody goat anti-mouse IgG-FITC (Santa Cruz Biotechnology, Santa Cruz, CA) at room temperature for 1 hour; or FITC-conjugated mouse anti-ZO-1 (clone ZO-1-1A12, Zymed Laboratories, Carlsbad, CA) at a 1:50 dilution in 1% Goat Serum (Vector Laboratories, Burlingame, CA). Sections were finally rinsed with PBS, covered with Vectashield mounting medium containing DAPI (Vector Laboratories, Burlingame, CA), and examined using a Zeiss Axio Observer Z1 motorized microscope with apotome (Thornwood, NY).

### Measurement of bladder tissue epithelial and total areas

Measurements of bladder cross sectional areas were determined from 2X digital images using a Nikon Eclipse TE300 Inverted Microscope with NIS-Elements BR 3.00 Imaging Software with custom macros (Melville, New York, USA). The two custom macros used were created to measure the area of each cell layer in the bladders [the epithelial layer (if present), the interstitial layer, and the muscle layer]; one custom macro was used for bladders that had an epithelial layer, while the other was used in denuded bladders.

### Statistical analysis

Epithelial area measurements were analyzed by analysis of variance (ANOVA). Because the variance of these measurements increased when the mean value increased, we performed a square root transformation to stabilize the variance, and performed two-way ANOVA (with treatment group and day being the two factors) on the transformed outcome variable. Post hoc comparisons of multiple groups were carried out using the Tukey-Kramer method. P < .05 was considered to indicate a significant difference between groups.

## Results

**Figure 1:**
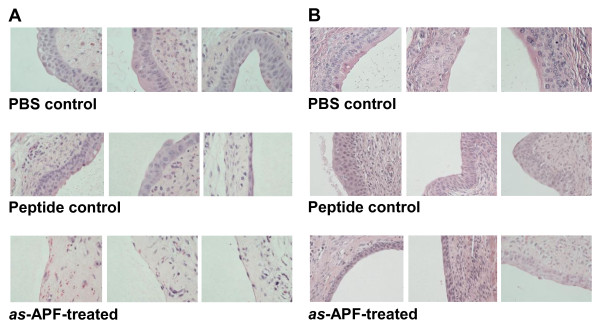
**Inhibition of bladder epithelial repair in CBA/J/Hsd mice following treatment with*as*-APF.** H&E stained bladder sections from mice treated with *as*-APF following bladder epithelial injury show inhibition of epithelial regeneration as compared to mice treated with PBS or inactive control nonglycosylated peptide. **A**) 3 days of *as-*APF treatment; **B**) 14 days of *as-*APF treatment. Representative data shown for the 3 mice in each treatment group from one experiment; experiment performed five times. (200X final magnification).

To determine whether the bladder epithelial thinning found in PBS/IC bladder epithelial cells both *in vivo* and/or *in vitro* might be caused by APF inhibition of epithelial repair, we determined whether daily 3 hour intravesical treatment with synthetic *as-*APF inhibited normal bladder epithelial repair in 5–6 week old female CBA/J/Hsd mice for 1, 2, 3, 4, 7, 14 or 21 days following injury with 3% acetic acid. For each experiment, 3 mice were treated with each of the 3 treatments (*as-*APF in PBS, an equimolar concentration of inactive unglycosylated APF peptide in PBS, or PBS vehicle alone). On days 1 and 2, there was no difference in the amount of epithelial cells between treatment groups, with all but one of the mice having no, or only one, layer of epithelial cells in all 3 groups (the remaining one mouse in the PBS control group had 3 layers of epithelial cells by day 2) (data not shown). However, as shown in Figure 1A, by day 3 differences in the number of epithelial cell layers were evident in the bladder of six week old female CBA/J/Hsd mice treated daily with *as-*APF following wounding with 3% acetic acid (0–1 layer of epithelial cells) as compared to controls treated with either inactive control unglycosylated APF peptide (1–4 layers of epithelial cells) or PBS carrier alone (3–4 layers of epithelial cells). In repeated experiments, all but one of the mice in both control groups had 2–4 layers of bladder epithelial cells by this day 3 time point, whereas most of the *as-*APF-treated mice had only 1–2 layers of epithelial cells, and the bladder wall remained denuded of epithelium at the same time point for 7 of the 15 total animals in this group, suggesting an inhibition of bladder epithelial repair by *as*-APF.

**Figure 2:**
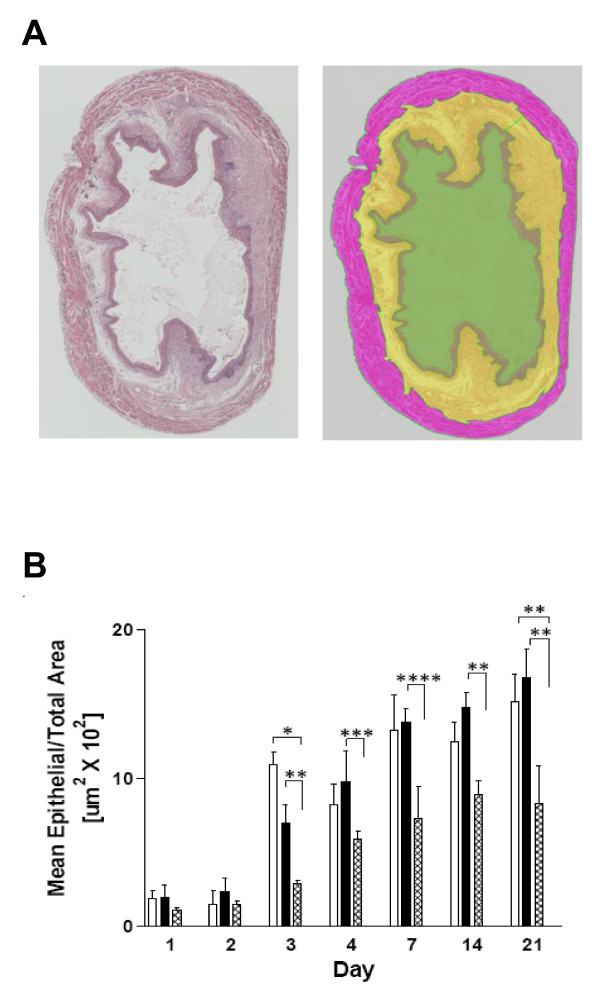
**NIS-Elements BR 3.00 software image conversion for quantification of total epithelial, subepithelial interstitial, and muscularis layers of mouse bladder sections.A**) Images of H&E-stained mouse bladder sections (shown on left) were generated at 20X using a Nikon Eclipse TE300 Inverted Microscope and the area of specific tissue layers measured using NIS-Elements BR 3.00 Imaging Software with custom macros (resulting image shown on right). **B**) These images were then used to determine the ratio of epithelial area to total bladder cross-sectional area for mice in each treatment group for all time points using NIS-Elements BR 3.00 Imaging Software. Data were combined from five experiments and analyzed using two-way ANOVA; *p < .0001; **p < .01; ***p < .02; ****p < .04. Treatment group: PBS □; inactive control peptide ■; *as-*APF ░.

**Figure 3:**
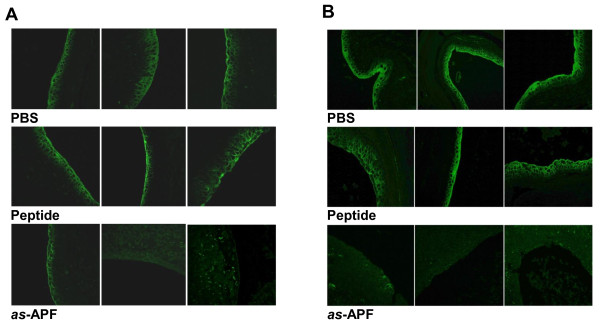
**Inhibition of uroplakin III (UPIII) expression in CBA/J/Hsd mouse bladders following treatment with*as*-APF.** Bladder sections from mice treated with *as*-APF following bladder epithelial injury show decreased UPIII immunofluorescence staining as compared to mice treated with PBS or inactive control nonglycosylated peptide. **A**) 7 days of *as-*APF treatment; **B**) 21 days of *as-*APF treatment. Representative data shown for the 3 mice in each treatment group from one experiment; experiment performed three times. (200X final magnification).

Bladder epithelial repair continued during the subsequent days of the experiment, with the number of epithelial cell layers generally increasing in all 3 treatment groups (images shown for day 14 in Figure 1B, with graphical results for all time points shown in Figure 2). However, while the number of epithelial cell layers in the *as*-APF-treated mice also increased from 0–1 on day 3 (Figure 1A) to 2–3 by day 14 (Figure 1B), these animals continued to have marked epithelial thinning as compared to mice in either the inactive peptide (3–6 cell layers) or PBS (4–6) control groups (Figure 1B), and the epithelium in the *as*-APF-treated mice did not increase further by day 21 (Figure 2).

Because the apparent number of epithelial cell layers can be influenced by bladder distension, digital images were also obtained for measurement of the bladder epithelium, subepithelial interstitium, and muscularis (Figure 2A), and combined data from five total experiments were analyzed by ANOVA (10–14 mice per group on days 1,2,3,7 and 14; 3–5 mice per group on days 4 and 21). As shown in Figure 2B, mice treated with *as-*APF had decreased epithelial area throughout the 21 days as compared to control mice, reaching significance vs. one or both control groups on days 3–21 (p < .05 for all groups by two-way ANOVA following the appropriate square root transformation of data for increasing variance). In comparison, there was no significant difference in the mean ratio of epithelial/total bladder cross sectional area between the two control groups throughout the experiment. Post hoc analyses indicated that the average of the transformed mean epithelial/total area over all measured days is significantly different between *as*-APF and PBS, as well as between *as*-APF and peptide treatment groups (p < 0.0001 for both comparisons), and confirmed that there was no significant difference between PBS and peptide treatment groups (p = 0.96).

In addition to bladder epithelial thinning, decreased bladder epithelial expression of uroplakin III (UPIII) and zonula occludens type 1 (ZO-1) has also been described in bladder tissue from IC/PBS patients [6,7]. Therefore, expression of both proteins in the regenerating bladder epithelium was also determined in *as*-APF-treated vs. control mice using immunofluorescence microscopy. While little UPIII expression was evident in animals from any of the treatment groups on days 1–4 (data not shown), mice treated with either control compound (inactive peptide or PBS) had increased UPIII expression in the superficial (more well-differentiated) bladder epithelial cells as compared to mice treated with *as*-APF by day 7 (Figure 3A); negative controls stained with an unrelated isotype control primary antibody or with secondary antibody alone did not show evidence for nonspecific background staining (data not shown). This difference in UPIII expression continued with mice in either control group having greatly increased expression as compared to *as*-APF-treated mice by day 21 (Figure 3B).

**Figure 4:**
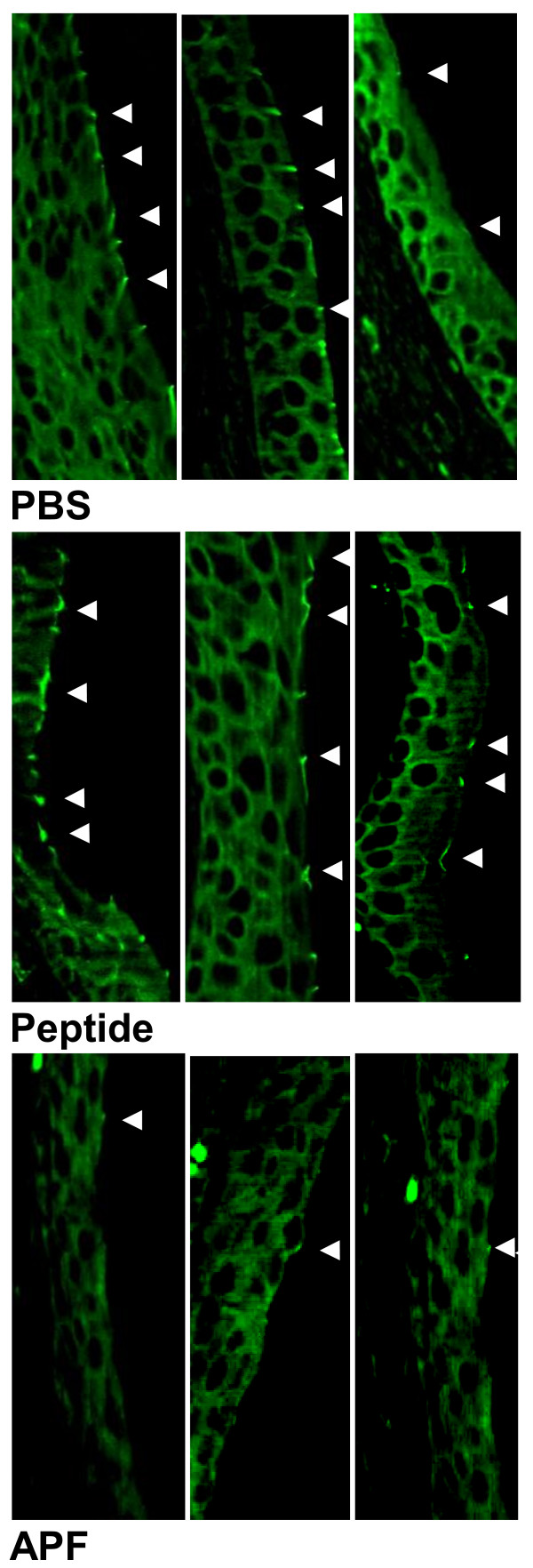
**Inhibition of zonula occludens type 1 (ZO-1) expression in CBA/J/Hsd mouse bladders following 14 days of treatment with*as*-APF.** Bladder sections from mice treated with *as*-APF following bladder epithelial injury show decreased ZO-1 immunofluorescence staining (shown by small junctions between cells indicated by arrows) as compared to mice treated with PBS or inactive control nonglycosylated peptide. Representative data shown for the 3 mice in each treatment group from one experiment; experiment performed three times. (500X final magnification).

ZO-1 expression (evident as a small distinct enhanced junctional area between cells in the terminally differentiated luminal cell layer) was not clearly seen in any mice in any treatment group up to and including day 7 of the experiment (data not shown). While such areas of enhancement were definitely evident in the regenerating epithelium from mice in all 3 treatment groups by day 14 (Figure 4), ZO-1 expression was also greater in the control mice treated with inactive peptide or PBS as compared to mice treated with *as*-APF. Negative controls stained with an unrelated FITC-labeled antibody did not show evidence for nonspecific background staining (data not shown).

## Discussion

In this manuscript we present evidence that mice treated with an active synthetic APF derivative [Galβ1-3GalNAcα-*O*-TVAAVVVA] have decreased bladder epithelial repair following intravesical acetic acid instillation. This finding mimics the bladder epithelial thinning/ulceration found in biopsies from IC/PBS patients [1-6] as well as the decreased proliferation seen in both bladder epithelial cells explanted from IC/PBS patients and *as*-APF-treated primary normal bladder epithelial cells [13,36]. In addition, our data indicate that *as*-APF treatment of these mice also resulted in decreased expression of UPIII and ZO-1 in the regenerating epithelium, similar to abnormalities in expression of these proteins found in bladder biopsy specimens from IC/PBS patients *in vivo*[6,7], as well as explanted epithelial cells from IC/PBS patients and *as*-APF-treated primary normal bladder epithelial cells *in vitro*[15].

Finding a reliably effective treatment for IC/PBS has been hampered for many years by the lack of a well-understood inducible animal model with histologic/biochemical features shared by the disease [22,35]. Many putative animal models for IC/PBS have been developed to date, but evidence for abnormal expression of the same bladder epithelial proteins as documented in human biopsy specimens is scant for these models. For example, although the feline IC model and the protamine sulfate rat model share evidence for decreased bladder epithelial tight junctions [51,52], only the feline IC model has been documented to have decreased ZO-1 expression and increased epithelial permeability [51]. Like bladder tissue from IC/PBS patients, the feline IC model also has been shown to have increased iNOS [23]. However, unlike patients with the human illness (who exhibit *in*creased P2X2 or P2X3 urothelial receptors) cats with feline IC have *de*creased bladder epithelial P2X1 receptor expression without abnormalities in P2X2 or P2X3 expression [53]. In addition, because the etiology of feline IC remains unknown it is not an inducible model, and because of its sporadic and spontaneous nature cats with feline IC can be difficult for many researchers to obtain.

Similarly, the relationship of the antigen-induced, virally-induced, chemical toxin-induced, or autoimmune models of cystitis to the human disease are also not clear, as there is no definite evidence for a similar pathogenesis (with a defined antigen, virus, toxin, or autoantigen) for the human disease [22]; therefore, although these models are more readily available, their utility for testing therapeutic or preventive agents for this syndrome also remains unknown. Abnormally expressed bladder epithelial cell genes similar to those found in bladder biopsies of IC/PBS patients have been limited to UPIII [decreased in acrolein-induced cystitis [54] and cyclophosphamide (CYP)-induced cystitis [55]], and iNOS [increased in the CYP model] [56]. However, bladder epithelial cell UPIII expression rapidly normalizes (within 24 hours) in the CYP-induced cystitis model, and bladder hyperreflexia in these models can similarly resolve within days [57] indicating the transient nature of some of these changes in these models. Therefore, additional studies are also required to compare the durability of abnormal bladder epithelial gene expression in all of these models for us to understand the relative utility of each model for studies of IC/PBS pathogenesis and treatment.

The model described in this preliminary report has certain advantages over the other animal models that have been described to date. It is an inducible rodent model, making it potentially more readily available than the spontaneous feline model. This mouse model also has changes in expression of two proteins similar to changes found consistently in patients with IC/PBS – UPIII and ZO-1. Unlike some protein expression abnormalities found in the other models to date, these changes appear to be durable (sustainable) for up to 3 weeks. And whereas most of the other models are induced by factors that are almost certainly not causative in the pathogenesis of IC/PBS, this model is based on a potential etiology of IC/PBS (APF) that was first discovered in urine from patients with this illness, subsequently shown to be made by bladder epithelial cells in these patients and to induce abnormal expression of at least six epithelial proteins known to be abnormally expressed in bladder tissue from IC/PBS patients [14-16,36]. Because APF inhibits bladder epithelial cell replication but does not by itself induce cell death *in vitro*[13,16] we previously hypothesized that APF may inhibit bladder epithelial repair following injury caused by other inciting factors such as a urinary tract infection [16,58]. However, the use of acetic acid to induce such bladder epithelial injury prior to APF instillation in the current model is also by itself not related to the pathogenesis of IC; future refinements to the model may therefore include induction of epithelial shedding by bacteria known to cause cystitis [48] prior to APF instillation.

Disadvantages of this model, however, include its requirement for daily intravesical instillations of active synthetic APF (or its control peptide), making it relatively labor-intensive and expensive for a rodent model. In addition, some of the variability seen in this model regarding the effects of APF on epithelial repair or gene expression may result from limited (only 3 hours daily) exposure to the instilled APF. However, based on the preliminary results from this model, development of a continuous APF release model (via an implanted pump), or a transgenic mouse model that would express active APF either constitutively or inducibly, appear to be warranted.

## Conclusions

Bladder instillation of *as*-APF inhibits bladder epithelial repair and expression of UPIII and ZO-1 in CBA/J mice following transurethral acetic acid infusion. Because bladder epithelial thinning, decreased UPIII expression, and decreased ZO-1 expression are consistently found in IC/PBS patient bladder epithelial biopsies, this model may be useful for studying the pathophysiology of this illness, and the effect of potential therapies.

## Abbreviations

APF, Antiproliferative factor; as-APF, Asialylated APF; FITC, Fluorescein isothiocyanate; H&E, Haematoxylin and eosin; IC, Interstitial cystitis; IC/PBS, Interstitial cystitis/painful bladder syndrome; PBS, Phosphate buffered saline; UPIII, Uroplakin III; ZO-1, Zonula occludens type 1.

## Competing interests

Susan Keay is named as an inventor on patents for the composition and use of APF (patents owned by the University of Maryland, Baltimore and the Department of Veterans Affairs).

## Authors’ contributions

SL performed the animal experiments, as well as tissue specimen fixation and immunofluorescence staining, plus light and immunofluorescence microscopy. AO and GC performed measurements of bladder cross sectional areas by light microscopy. MZ performed the statistical analysis. SK and DJ supervised the performance of the experiments, data analysis and preparation of the manuscript. All authors read and approved the final manuscript.

## Pre-publication history

The pre-publication history for this paper can be accessed here:

http://www.biomedcentral.com/1471-2490/12/17/prepub
